# #CDCGrandRounds and #VitalSigns: A Twitter Analysis

**DOI:** 10.29024/aogh.2381

**Published:** 2018-11-05

**Authors:** Ashley M. Jackson, Lindsay A. Mullican, Jingjing Yin, Zion Tsz Ho Tse, Hai Liang, King-Wa Fu, Jennifer O. Ahweyevu, Jimmy J. Jenkins, Nitin Saroha, Isaac Chun-Hai Fung

**Affiliations:** 1 Department of Epidemiology and Environmental Health Sciences, Jiann-Ping Hsu College of Public Health, Statesboro, GA 30460, US; 2 Department of Biostatistics, Jiann-Ping Hsu College of Public Health, Statesboro, GA 30460, US; 3 College of Engineering, The University of Georgia, Athens, GA 30062, US; 4 School of Journalism and Communication, The Chinese University of Hong Kong, HK; 5 Journalism and Media Studies Centre, The University of Hong Kong, HK; 6 MIT Media Lab, Massachusetts Institute of Technology, Cambridge, MA 02139, US; 7 Department of Computer Science, The University of Georgia, Athens, GA 30062, US

## Abstract

**Background:**

The CDC hosts monthly panel presentations titled ‘Public Health Grand Rounds’ and publishes monthly reports known as Vital Signs. Hashtags #CDCGrandRounds and #VitalSigns were used to promote them on Twitter.

**Objectives:**

This study quantified the effect of hashtag count, mention count, and URL count and attaching visual cues to #CDCGrandRounds or #VitalSigns tweets on their retweet frequency.

**Methods:**

Through Twitter Search Application Programming Interface, original tweets containing the hashtag #CDCGrandRounds (n = 6,966; April 21, 2011–October 25, 2016) and the hashtag #VitalSigns (n = 15,015; March 19, 2013–October 31, 2016) were retrieved respectively. Negative binomial regression models were applied to each corpus to estimate the associations between retweet frequency and three predictors (hashtag count, mention count, and URL link count). Each corpus was sub-set into cycles (#CDCGrandRounds: n = 58, #VitalSigns: n = 42). We manually coded the 30 tweets with the highest number of retweets for each cycle, whether it contained visual cues (images or videos). Univariable negative binomial regression models were applied to compute the prevalence ratio (PR) of retweet frequency for each cycle, between tweets with and without visual cues.

**Findings:**

URL links increased retweet frequency in both corpora; effects of hashtag count and mention count differed between the two corpora. Of the 58 #CDCGrandRounds cycles, 29 were found to have statistically significantly different retweet frequencies between tweets with and without visual cues. Of these 29 cycles, one had a PR estimate < 1; twenty-four, PR > 1 but < 3; and four, PR > 3. Of the 42 #VitalSigns cycles, 19 were statistically significant. Of these 19 cycles, six were PR > 1 and < 3; and thirteen, PR > 3.

**Conclusions:**

The increase of retweet frequency through attaching visual cues varied across cycles for original tweets with #CDCGrandRounds and #VitalSigns. Future research is needed to determine the optimal choice of visual cues to maximize the influence of public health tweets.

## Introduction

Social media is an important platform for public health communication [1]. Topics of great relevance to global health, such as avian influenza, Ebola, HIV, malaria, Middle East respiratory syndrome, tuberculosis, and Zika, were discussed on Twitter and other social media platforms [2,3,4,5,6,7,8,9]. In the United States, federal, state, and local health agencies use social media to share and disseminate health-related information to the general public [10,11,12]. In particular, the Centers for Disease Control and Prevention (CDC) maintained a portfolio of social media communication efforts [13]. Prior research has studied user engagement of CDC Facebook communication during the Ebola emergency response [14,15], as well as individual Twitter chats that CDC hosted during the Ebola and Zika outbreaks [16,17]. However, no studies to date analyze how CDC Twitter communication promoted their monthly events and publications. In this case study, we are going to focus on CDC Public Health Grand Rounds and CDC Vital Signs and their related Twitter communication.

The CDC hosts a monthly panel presentation coupled with a webcast titled ‘Public Health Grand Rounds’ that has been in circulation since September of 2009. This monthly webcast consists of a panel of speakers who present the latest scientific research and public health advice pertinent to a selected topic. Intended audience includes both healthcare professionals and members of the lay public. The goal of Public Health Grand Rounds is to facilitate discussions about the potential impacts of public health issues through presenting scientific evidence and updated research. These discussions focus on how to solve public health challenges and develop recommendations for future research [18].

Vital Signs is a CDC monthly report which is intended to provide subscribers with information regarding health issues. It was launched in 2010. The report includes an early release of the *Morbidity and Mortality Weekly Report* (MMWR), which is also a CDC publication, a graphic fact sheet and website, a media release, and social media tools. The report is released on the first Tuesday of every month and is available to anyone who signs up on the CDC website. Every report follows a different topic. The topics include alcohol, cancer, cardiovascular diseases, food safety, prescription drug overdoses, teen pregnancy, tobacco, healthcare-associated infections, HIV/AIDS, motor vehicle safety, obesity, and others [19].

To promote Public Health Grand Rounds and Vital Signs, the CDC disseminates related information to their Twitter followers using the hashtags #CDCGrandRounds and #VitalSigns, respectively. In this study, we analyzed two corpora of tweets, each with one of these two hashtags.

To increase engagement with their followers, CDC health communication specialists often attach visual cues, such as images or videos, to their tweets. In a recent study, it was found that attaching visual cues to posts posted by federal health agencies on their Facebook pages would generate more engagement with Facebook users [10]. In this study, we aimed to answer two research questions: (a) to quantify the effect of hashtag count, mention count, and URL link count on retweet frequency between the Twitter corpora of #CDCGrandRounds and #VitalSigns respectively; and (b) to quantify the effect of attaching visual cues on retweet frequency across cycles of #CDCGrandRounds and #VitalSigns Twitter health communication.

## Methods

*Data collection.* We retrieved tweets’ IDs via web scraping and then used Twitter Search Application Programming Interface to download the tweet’s meta-data to a server at Athens, Georgia, USA. Details of our data retrieval methods can be found in the Online Supplementary Materials.

*Data sets.* All 6,966 original tweets containing the hashtag #CDCGrandRounds dated from April 21, 2011 to October 25, 2016 were retrieved. In this corpus, four tweets were posted on April 21, 2011 and were excluded from further analysis. The rest of the corpus (N = 6,962) began with August 18, 2011 and was the basis of subsequent analysis. All original tweets containing the hashtag #VitalSigns dated from March 19, 2013 to October 31, 2016 were retrieved (N = 15,015).

*Analysis of #CDCGrandRounds and #VitalSigns data sets.* Descriptive statistics was reported, and the respective 10 most frequent Twitter users (Twitter handles) and URL links of the #CDCGrandRounds and #VitalSigns corpora were identified. Univariable and multivariable negative binomial regression models were applied to the two corpora to test if the three variables of interest (hashtag count, mention count, URL count) were associated with retweet frequency, after controlling for four confounders (users’ followers count, friends’ count, status count, and favorite count.)

*Analysis by cycle*. Each corpus was sub-set into cycles (#CDCGrandRounds: n = 58, #VitalSigns: n = 42). We designated each cycle chronologically starting from ‘1’. For the #CDCGrandRounds corpus, a cycle was defined as all tweets referring to the specified topic of the specific Public Health Grand Round event. Because CDC might promote a Public Health Grand Round event ahead of time, and tweets might continue to be retweeted after the next event, there was no clear definition for when each cycle started and ended. The dates were obtained by manually reading and grouping the tweets by similar content. Tweets that fell within a cycle corpus but were unrelated to the topic were excluded. The first cycle in the #CDCGrandRounds corpus was ‘Newborn Screening: Improving Outcomes’ in August 2011, and our data set ended with the cycle on ‘Changes in Clinical Diagnostics and Tracking Infectious Diseases’ in October 2016.

For the #VitalSigns corpus, a cycle was defined as the first day of the publication release, which was the first Tuesday of each month, until the day before the next publication was released. Any tweets that were not related to the assigned Vital Sign publication were excluded. Our #VitalSigns corpus began with the March 2013 cycle, ‘Making Health Care Safer – Stop Infections from Lethal CRE Germs Now’ and ended with the October 2016 cycle on ‘Dental Sealants Prevent Cavities’.

Manual coding was then performed on the top 30 most influential tweets, defined as the 30 tweets with the highest number of retweets for each cycle. These were identified in each cycle and manually coded as either containing a form of visual cues (as ‘1’) or not (as ‘0’). ‘Visual cues’ here was defined as a still image or a video.

Univariable negative binomial regression models were applied to the sub-corpus of each cycle to compute the prevalence ratio (PR) of retweet frequency between tweets with and without visual cues. Multivariable regression models were not applied because of the small sample size of 30 manually coded tweets of each cycle.

*Statistical language.* R, version 3.3.1 [20], was used via RStudio, version 0.99.903 [21] to perform all analyses.

*Ethics statement.* This project was approved by the Institutional Review Board (IRB) of Georgia Southern University (H15083) and was determined to be exempt from full review under the exemption category B2.

## Results

Table 1 presents the descriptive statistics of both corpora of #CDCGrandRounds and #VitalSigns tweets. We found that 99% of #CDCGrandRounds tweets and 89% of #VitalSigns tweets were categorized as English by Twitter (Table 1). In the #CDCGrandRounds corpus, the top 10 users were all CDC users and the top three URL domains were CDC, Twitter, and YouTube (Table 2). In the #VitalSigns corpus, @CDCgov and @DrFriedenCDC ranked top number 3 and 5 users respectively and there were 2,058 (18.49% of 11,129) URL links directed to CDC’s domain (www.cdc.gov). URL links from Twitter and Instagram were top number 1 and 5 respectively. The @RedneckJournal and @Pirate_journal were the top two Twitter users who posted #VitalSigns tweets and the frequencies of URL links directed to their domains ranked third and fourth respectively.

**Table 1:** Descriptive statistics of #CDCGrandRounds and #VitalSigns.

	#CDCGrandrounds		#VitalSigns

**Total number of original tweets in the corpus**	6,962	*	15,015
**Time Frame**	August 18, 2011 to October 25, 2016		March 19, 2013 to October 31, 2016
**English tweets, n (%)**	6875 (98.75)		13401 (89.25)
**Number of unique users**	1055		5154
**Tweets with 0 URL links (%)**	4695 (67.44)		5019 (33.43)
**Tweets with 1 URL links (%)**	2118 (30.42)		8873 (59.09)
**Tweets with 2 URL links (%)**	146 (2.10)		1113 (7.41)
**Tweets with 3 URL links (%)**	3 (0.04)		10 (0.07)
**Number of cycles in the sample**	58		42
**Number of original tweets per cycle, frequency**^†^			
**Range**	6–349		151–1628
**Median (Inter-quartile range)**	119 (75–157.5)		256 (218.75–306.75)
**Mean (standard deviation)**	119.5172 (74.17056)		368.125 (322.4237)

*We excluded 4 #CDCGrandRounds tweets that were posted on April 21, 2011. These 4 tweets were not included in any of the cycles of the #CDCGrandRounds corpus. ^†^For the frequency of the cycles of #VitalSigns, we excluded Cycle 1 and Cycle 42 here.

**Table 2:** Top 10 users (Twitter handles) and top 10 URL domains in the corpora of #CDCGrandRounds and #VitalSigns respectively.

#CDCGrandRounds	N = 6,962 tweets	#VitalSigns	N = 15,015 tweets

Twitter handle	Frequency (%)	Twitter handle	Frequency (%)

@CDCgov	1381 (19.8)	@RedneckJournal	759 (5.05)
@CDC_eHealth	882 (12.7)	@Pirate_Journal	467 (3.11)
@CDCInjury	349 (5.0)	@CDCgov	389 (2.59)
@CDCChronic	212 (3.0)	@CommFdnsCanada	260 (1.73)
@MillionHeartsUS	211 (3.0)	@DrFriedenCDC	245 (1.63)
@CDC_Cancer	138 (2.0)	@LdnCommFdn	203 (1.35)
@DrFriedenCDC	136 (2.0)	@HamCommFdn	113 (0.75)
@CDCSTD	135 (1.9)	@CDC_eHealth	112 (0.75)
@CDC_NCBDDD	126 (1.8)	@GoldenComFdn	109 (0.73)
@CDC_HIVAIDS	102 (1.5)	@Oxehealth	90 (0.60)
**#CDCGrandRounds**	**N = 2,419 URL links**	**#VitalSigns**	**N = 11,129 URL links**

**URL domain**	**Frequency (%)**	**URL domain**	**Frequency (%)**

www.cdc.gov	1424 (58.87)	Twitter.com	2670 (23.99)
Twitter.com	398 (16.45)	www.cdc.gov	2058 (18.49)
www.youtube.com	160 (6.61)	www.redneckjournal.com	658 (5.91)
Storify.com	34 (1.41)	www.piratejournal.com	467 (4.20)
www.onlinevideoservice.com	25 (1.03)	www.instagram.com	416 (3.74)
Wm.onlinevideoservice.com	21 (0.87)	www.youtube.com	319 (2.87)
Paper.li	18 (0.74)	www.vitalsignscanada.ca	157 (1.41)
www.facebook.com	12 (0.50)	www.facebook.com	133 (1.20)
Millionhearts.hhs.gov	10 (0.41)	www.whizart.com	130 (1.17)
www.hhs.gov	9 (0.37)	Soundcloud.com	102 (0.92)
*URL links not resolved*	*54 (2.23)*	*URL links not resolved*	*406 (3.65)*

In both corpora of #CDCGrandRounds and #VitalSigns tweets, all three predictor variables of interest and the four potential confounders were found to be significant in the univariable analyses, and they were entered into the multivariable negative binomial regression model (Table 3). For the #CDCGrandRounds corpus, after controlling for users’ followers count, friends’ count, status count, and favorite count, it was found that a unit increase in the number of hashtag will increase the retweet frequency by 8% (adjusted prevalence ratio, aPR = 1.0784, 95% CI, 1.0302, 1.1292, p < 0.001); increasing mention by one will reduce the retweet frequency of 14% (aPR = 0.8613, 95% CI, 0.8152, 0.9100; p < 0.001); and increasing URL link count by one will increase the retweet frequency by 78% (aPR = 1.7773, 95% CI, 1.6729, 1.8890; p < 0.001) (Table 3). For the #VitalSigns corpus, after controlling for users’ followers count, friends’ count, status count, and favorite count, it was found that adding one more hashtag would decrease retweet frequency by 3% (aPR = 0.9688, 95% CI, 0.9472, 0.9908, p < 0.001), while adding one more mention and one more URL link would increase retweet frequency by 17% (aPR = 1.1748, 95% CI, 1.1404, 1.2104, p < 0.001) and 22% (aPR = 1.2155, 95% CI, 1.1587, 1.2752, p < 0.001) respectively.

**Table 3:** Prevalence ratio of retweet frequency in the corpora of #CDCGrandRounds and #VitalSigns tweets for predictor variables in univariable and multivariable negative binomial regression models.

	Univariable regression		Multivariable regression	

	Prevalence ratio (95% CI)	*P*-value	Prevalence ratio (95% CI)	*P*-value

**#CDCGrandRounds**				

Hashtag count	0.8208 (0.7790, 0.8657)	<0.001	1.0784 (1.0302, 1.1292)	<0.001
Mention count	0.6673 (0.6271, 0.7105)	<0.001	0.8613 (0.8152, 0.9100)	<0.001
URL link count	1.7273 (1.6045, 1.8608)	<0.001	1.7773 (1.6729, 1.8890)	<0.001
Log(follower count)*	1.3876 (1.3709, 1.4045)	<0.001	1.4332 (1.4111, 1.4557)	<0.001
Log(friend count)*	0.7996 (0.7711, 0.8296)	<0.001	0.9082 (0.8755, 0.9422)	<0.001
Log(status count)*	1.5522 (1.4939, 1.6130)	<0.001	0.8538 (0.8213, 0.8878)	<0.001
Log(favorite count)*	0.9424 (0.9214, 0.9637)	<0.001	1.0805 (1.0579, 1.1035)	<0.001
**#VitalSigns**				

Hashtag count	0.6866 (0.6654, 0.7085)	<0.001	0.9688 (0.9472, 0.9908)	0.005
Mention count	0.7411 (0.7114, 0.7726)	<0.001	1.1748 (1.1404, 1.2104)	<0.001
URL link count	3.7244 (3.4586, 4.0113)	<0.001	1.2155 (1.1587, 1.2752)	<0.001
Log(follower count)*	1.7855 (1.7642, 1.8073)	<0.001	2.0256 (1.9957, 2.0563)	<0.001
Log(friend count)*	0.8634 (0.8309, 0.8975)	<0.001	0.8583 (0.8376, 0.8795)	<0.001
Log(status count)*	1.1712 (1.1324, 1.2106)	<0.001	0.6579 (0.6441, 0.6719)	<0.001
Log(favorite count)*	1.1475 (1.1188, 1.1766)	<0.001	1.1205 (1.1043, 1.1370)	<0.001

*To perform the logarithmic transformation, the frequency of follower count, friend counts, status count and favorite count were added 0.5 respectively to avoid the situation of having log(0). CI, confidence interval.

Tables S1 and S2 in the Online Supplementary Materials present the top retweet for each cycle of the #CDCGrandRounds and #VitalSigns corpora respectively, and whether they contain visual cues, as well as their retweet frequency.

**Figure 1:**
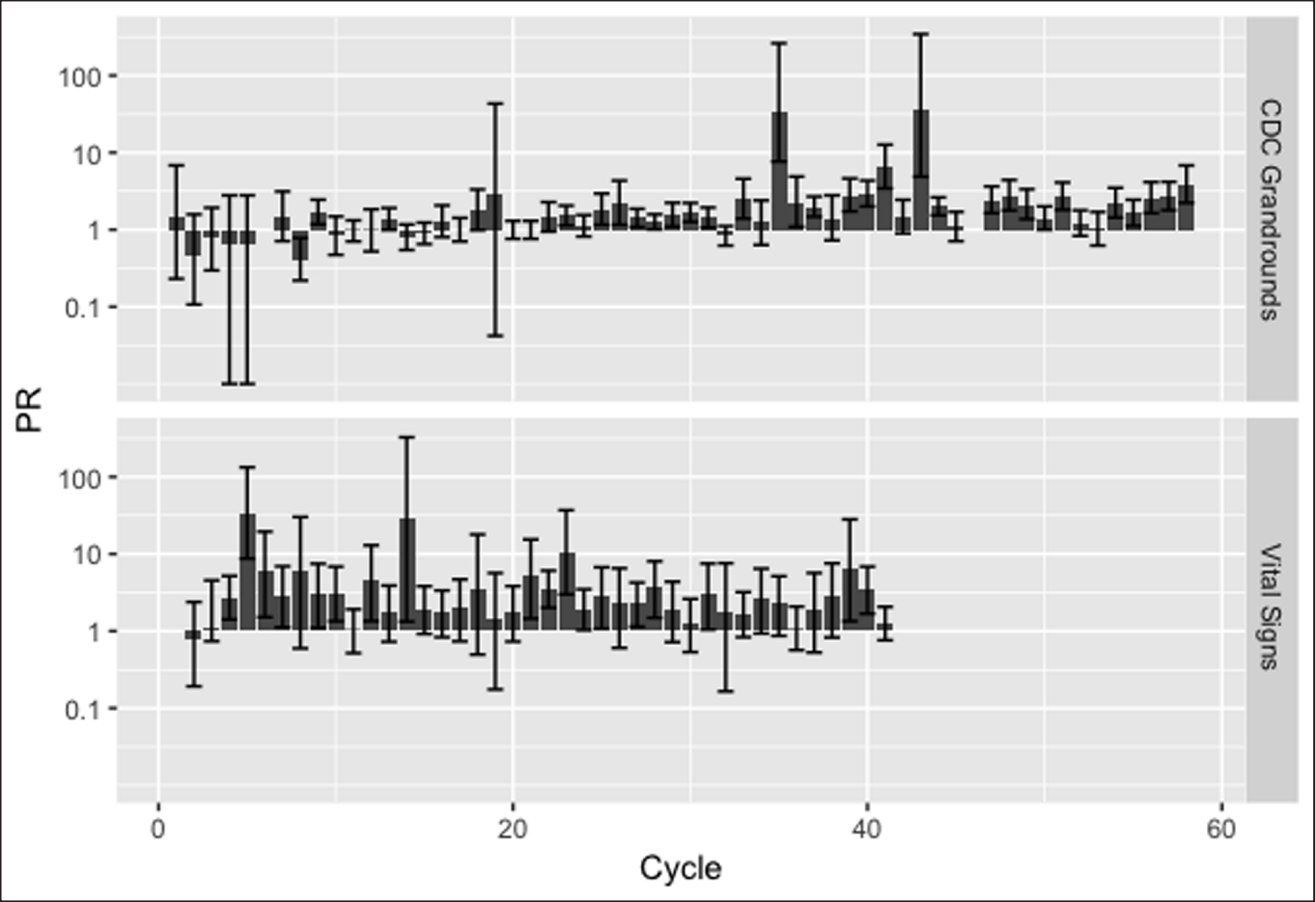
Prevalence ratio (PR, with 95% confidence intervals) of the effect of attaching a photo or video on retweet count for #VitalSigns over different cycles.

Figure 1 presents the per-cycle probability ratios of retweets for original tweets with images or videos as compared to those without. Detailed results are presented in Tables S3 and S4 in the Online Supplementary Materials. Here we highlight a few important findings.

Of the 58 #CDCGrandRounds cycles, 29 were found to have statistically significant difference between tweets with and without visual cues (Table S3). Of these 29 cycles, one had a PR estimate < 1; twenty-four had PR between 1 and 3; and four had PR > 3. Two cycles were outliers: ‘Preventing Suicide: A Comprehensive Public Health Approach’ (September 2015) with PR = 36.353 (95% confidence intervals, CI, 4.869–343.845, P < 0.001) and ‘Understanding the Causes of Major Birth Defects: Steps to Prevention’ (January 2015) with PR = 34.713 (95% CI, 7.662–261.591, P < 0.001).

Of the 42 #VitalSigns cycles, 19 were statistically significant (Table S4). The PR estimates of six of these 19 cycles were between 1 and 3; and for 7 cycles, PR were between 3 and 5; for 6 cycles, PR was > 5. There were three outliers: ‘Prescription Painkiller Overdoses’ (July 2, 2013) with PR = 33.514 (95% CI, 8.715, 133.357, P < 0.001), ‘Preventing Norovirus Outbreaks’ (June 3, 2014) with PR = 29.536 (95% CI, 1.330, 326.283, P = 0.007), and ‘Trucker Safety’ (March 3, 2015), with PR = 10.270 (95% CI, 2.992, 37.010, P < 0.001).

## Discussion

In this study, we quantified the strength of correlation between (a) hashtag count, mention count, and URL count, and (b) attaching visual cues to a CDC tweet and the retweet frequency. We used tweets promoting CDC Public Health Grand Rounds and Vital Signs as a case study.

Our analysis of the entire corpora of #CDCGrandRounds and #VitalSigns found that the presence of URL links increased retweet frequency in both corpora. These URL links could be links to images or videos. They could also be links to other sources of information, for example, websites or other social media posts. Our results confirm that links to visual cues or additional information sources would attract more engagement from the users.

However, for the effect of hashtag count and mention count on retweet frequency, our results from the two corpora were found to be of opposite direction. Hashtag count was associated with more retweets among #CDCGrandRounds tweets and with fewer retweets among #VitalSigns tweets. Mention count was associated with fewer retweets among #CDCGrandRounds tweets and with more retweets among #VitalSigns tweets. Our results suggest that the effect of hashtags and mentions on retweet frequency could be modified by contents or topics of the tweets.

In our per-cycle analysis, it was found that for #CDCGrandRounds, the attachment of visual cues increased the probability of the tweets being retweeted in 28 of 58 cycles, of which for 24 cycles, the PR was between one and three; for one cycle, between three and five and for three cycles, above five. For #VitalSigns, the attachment of a still image or video increased the probability of the tweets being retweeted in 19 of 42 cycles, of which for six cycles, the PR was between one and three; for seven cycles, between three and five; and for 6 cycles, above five. Our research is in line with a study of federal health agencies’ Facebook posts where the researchers found that visual cues increased Facebook posts engagement from users. In their study, photos and videos increase engagement by 6.253 and 2.833 folds respectively [10].

The health communication policy implication is clear: attaching visual cues to tweets helps improve retweet frequency at large. Nevertheless, while attaching visual cues to a CDC tweet increased retweets in nearly one half of the cycles of #CDCGrandRounds tweets and #VitalSigns tweets studied, such effect was not observed in the other half. It raises questions regarding whether the types of health contents may interact with the attachment of visual cues. However, since the confidence intervals of most estimates overlap with each other, we chose not to perform a multivariable analysis, as it would not be informative. Besides, there were many cycles and there would be many levels including each cycle and its interaction with visual cues. This would require estimating many parameters and our sample size is not large enough for that. Given the diversity of topics here, we could not identify with confidence specific topics that attaching images or videos may have an effect. In the future, control experiments could be performed to test the hypothesis that content might interact with visual cues to attract more retweets.

There are several limitations in this study. The frequency of retweets was obtained at a specific time point (cross-sectional), and thus we cannot trace the change of frequency of retweets over time. The hashtag #VitalSigns has been used by more than one organization to refer to different things. This is not specific to the Vital Signs reports of the CDC. Therefore, for multiple cycles, we had to manually code more than 30 tweets to obtain a sample of 30 relevant tweets with the highest frequency of retweets. Results of the effect of visual cues reported here are univariable PRs. Given the limited sample size and the nature of our sample, we did not control for the effect of other variables. Future studies can study other factors that can contribute towards increasing retweet frequency of tweets posted by health agencies.

## Conclusions

In this paper, we studied the effect of hashtag count, mention count, URL count, and attaching visual cues to tweets pertinent to CDC Public Health Grand Rounds and Vital Signs. URL links were associated with higher retweet frequency for both corpora while the effect of hashtag count and mention count differed between corpora. We found that there was an effect at large, of increasing retweets by attaching visual cues, but the effect varied depending on the cycles. In line with current practice by CDC health communicators, we recommend the continuation of attaching visual cues to tweets to increase their engagement with Twitter users who follow CDC Twitter accounts.

## Additional File

The additional file for this article can be found as follows:

10.29024/aogh.2381.s1 Online Supplementary Materials. Data Extraction and Processing.
