# Supplementary material for: #CDCGrandRounds and #VitalSigns: A Twitter Analysis
Source: Ann Glob Health. 2018 Nov 5;84(4):710–6. doi: 10.29024/aogh.2381 (PMC6748269; doi:10.29024/aogh.2381)
Supplement: Online Supplementary Materials. — Data Extraction and Processing. [file agh-84-4-2381-s1.pdf]

Online Supplementary Materials

Data Extraction and Processing

Limitations on using Twitter Search Application Programming Interface (API) to retrieve tweets. While Twitter Search API could be used to retrieve tweets with a particular hashtag, we did not use that method directly. The reasons were as follows. Twitter Search API allows queries against the indices of recent or popular tweets and behaves similarly to, but not exactly like the Search feature available in Twitter mobile or web clients, such as Twitter.com search. Twitter Search API is based on relevance but not on completeness. Furthermore, Twitter sets restrictions on how old one can grab the data from its API. Twitter Search API searches against a sampling of recent Tweets published in the past 7 days.[1] Twitter also sets a limit on the number of requests one can make in a time period. Twitter allows the user only 180 requests per 15-minute window.[2] Thus, the process of tweet extraction is limited by these limits set by Twitter.

Web scraping to retrieve tweets' ID. To retrieve Twitter data older than two weeks, we relied on web scraping. With Twitter Advanced Search, we can read all the tweets with a particular hashtag, person, place or between dates by scrolling through the web page. The most important thing a tweet contains is a tweet ID; if one can get a tweet ID of an old tweet we can use the search API to get all the information of that tweet. On the Twitter website using Twitter Advanced Search, users can scroll as much as they want and read the tweets even as old as five years old.

Therefore, we performed Twitter scrapping to retrieve tweets older than one week. The first step was to obtain the IDs of tweets with a hashtag by scrolling automatically through the page using

TwitterScrapper, a Python library.[3] Given the date we can retrieve all tweet IDs with that hashtag from that date until present or any interval specified by the user.

Twitter Search API to retrieve meta-data. After obtaining the tweet IDs, we used Twitter Search API to retrieve all the metadata of that tweet. We made 180 requests every 15 minutes. The data retrieved was in JSON format. The data retrieval was completed and the data delivered to the analysts on November 13, 2016.

Data processing. The Twitter data was converted from JSON format into CSV format. The data was then processed in R. The retrieved contained all original tweets and some retweets. We kept only the original tweets by removing the retweets in the corpus through identifying any tweets with “RT” in the text. The #CDCGrandRounds contained 7,879 tweets prior to retweet removal and 6,966 tweets after retweet removal. The #VitalSigns contained 16,021 tweets prior to retweet removal and 15,015 tweets after retweet removal. The retweet frequency used in the data analysis is based on the meta-data of the original tweet. The original tweets in the #CDCGrandRounds data set were dated from April 21, 2011 to October 25, 2016. The original tweets containing the hashtag #VitalSigns were dated from March 19, 2013 to October 31, 2016.

References

1. Twitter. The Search API. 2017; https://dev.twitter.com/rest/public/search. Accessed June 20, 2017.
2. Twitter. GET serach/tweets. 2017; https://dev.twitter.com/rest/reference/get/search/tweets. Accessed June 20, 2017.
3. Taspinar A. TwitterScraper. 2017; https://github.com/taspinar/TwitterScraper. Accessed April 26, 2017.

Table S1. The tweet with the highest number of retweets for each cycle of #CDCGrandRounds, and whether it contained a visual cue (an image or a video).

**Date of Tweet (m/d/y)**	**Twitter User**	**Tweet Body**	**Visual cue (Yes/ No)**	**Retweet Frequency**
8/24/2011	CDCgov	Recorded CDC Public Health Grand Rounds on newborn screening is now available #NCBDDD #CDCGrandRounds http//tco/WFdSo2Q'	Y	11
9/27/2011	CDCgov	Recorded CDC Public Health Grand Rounds on Reducing Severe Traumatic Brain Injury in the US #CDCGrandRounds http//tco/p622DC6B'	Y	12
1/17/2012	CDCgov	'Watch the next #CDCGrandRounds on äóìThe Science Base for Prevention of Injury and Violenceäó • today at 1 pm ET http//tco/73G7PB7r'	Y	8
2/21/2012	MillionHeartsUS	#CDCGrandRounds 37 million Americans w/ hypertension do not have their blood pressure under control'	N	22
3/13/2012	CDCgov	Watch a live webcast of #CDCGrandRounds on Preventing Excessive Alcohol Use on March 20th at 1pm ET http//tco/YJHR0og3'	Y	15
5/15/2012	CDCgov	'Watch #CDCGrandRounds live webcast on multidrugresistant gonorrhea today at 1 pm ET and earn continuing education http//tco/JpvLbGGq'	Y	10
6/12/2012	CDCgov	About 1 in 4 women & 1 in 7 men have experienced physical violence by an intimate partner #CDCGrandRounds http//tco/tTN8ID1M'	Y	25
7/24/2012	CDCTobaccoFree	54 million people die each year due to tobacco related illnesses Watch #CDCGrandRounds live webcast today at 1pm EST'	N	59
8/21/2012	CDCgov	'There are over 11 million people living with HIV in the US Watch #CDCGrandRounds live webcast today at 1pm ET http//tco/KrVVi5vA'	Y	28
9/18/2012	CDCgov	'Diseases New to Minnesota Rocky Mountain Spotted Fever Powassan encephalitis Naeglari fowleri #CDCGrandRounds'	N	17
10/16/2012	CDCgov	Protective factors for SIDS include roomsharing w/o bedsharing breastfeeding pacifier use and being immunized #CDCGrandRounds'	N	26
11/13/2012	CDCgov	Be aware In recent survey of drs & nurses some admitted they sometimes or always reuse a syringe on a second patient #CDCGrandRounds'	N	48
12/13/2012	CDC_NCBDDD	'Obesity = common public health concern Affects those w/disabilities too Learn more at #CDCGrandRounds Tues 1pmET http//tco/kIm1DlHc'	Y	24
1/14/2013	CDCgov	Join the conversation 1/15 1pm EST with @DrGrosseCDC for #CDCGrandRounds Preventing Venous Thromboembolism http//tco/vZhrRE98'	Y	20

2/19/2013	CDCgov	'There are approx 26K HPVattributable cancers 21K of those are vaccine preventable #CDCGrandRounds'	N	24
3/19/2013	CDC_eHealth	'Join CDC for 'Reducing Teen Pregnancy in the US' at 1pm ET Watch webcast or follow live tweets at #CDCgrandrounds http//tco/DZ2ZjiBsCc'	Y	20
4/16/2013	CDCgov	'April is Minority Health Month Learn more about CDCäó»s work in reducing health disparities at http//tco/efSAfrwd5R #CDCGrandRounds'	N	28
5/23/2013	CDCgov	Did you miss the Hypertension Detect Connect Control webcast this week? Watch the #CDCGrandRounds video here http//tco/uqNN6AVMWJ'	Y	19
7/16/2013	CDC_Cancer	Dr Brawley 15K-20K lives per year could be saved in US if there was efficient colorectal #cancer screening & treatment #CDCGrandRounds'	N	20
9/19/2013	CDCgov	Did you miss the #CDCGrandRounds webcast on how technology can promote healthy living? Watch the video here http//tco/7MRdEVdaO4'	Y	15
11/13/2013	DrFriedenCDC	'Don't miss the next #CDCGrandRounds on alarming problem of antibiotic resistance Watch live Tuesday 11/19 1PM EST http//tco/sGyallw8jA'	Y	44
12/5/2013	CDCgov	'Did you miss the CDC webcast about Advanced Molecular Detection? Watch the video here http//tco/2dwlPhs78w #CDCGrandrounds'	Y	14
12/17/2013	DrFriedenCDC	As a consumer you don't have to remember to do anything to benefit from water fluoridation just drink tap water #CDCGrandRounds'	N	21
1/28/2014	CDCgov	Nanotechnology from Science Fiction to Real Life! Watch the new Beyond the Data #CDCGrandRounds http//tco/panGKSrlF4'	Y	18
2/25/2014	CDCInjury	Parents & communities can work together to prevent youth violence Watch new Beyond the Data video http//tco/dBLL893e9J #cdcgrandrounds'	Y	18
3/20/2014	CDCgov	'Did you miss the CDC webcast MultidrugResistant Tuberculosis? Watch the video here http//tco/qr5QKwT7Gd #CDCGrandrounds'	Y	19
4/18/2014	CDCgov	Dont miss #CDCGrandrounds session 4/22 1pm ET on autism spectrum disorder & evidencebased interventions http//tco/X7AquIITBA'	Y	20
5/27/2014	CDCgov	'Watch the new #CDCGrandRounds Beyond the Data video with CDC experts on using PrEP for prevention of HIV http//tco/BusdTJWJez'	Y	26
6/17/2014	CDCgov	'Follow @CDCgov TODAY at 1pm ET for live tweeting of #CDCGrandRounds session on #hepatitis C virus (HCV) http//tco/x1JEgCEfHa'	Y	36
8/19/2014	CDCgov	Warner #Infertility affects both women & men 6% of women ages 15-44 & 9% of men experience infertility #CDCGRandRounds'	N	20

9/22/2014	MillionHeartsUS	New #CDCGrandRounds Beyond the Data video preventing heart attacks & strokes w/ Dr John Iskander & Dr Janet Wright http//tco/KVcqSYGglP'	Y	18
10/21/2014	CDCgov	'Follow @CDC_eHealth TODAY at 1pm ET for live tweeting of #CDCGrandRounds session http//tco/ZiEuxrGvAB'	N	47
11/18/2014	CDC_eHealth	Follow @CDC_NCEZID today at 1pm ET for live tweeting of #CDCGrandRounds session on transplanttransmitted infections http//tco/7i7GBKtdtm'	Y	11
12/16/2014	CDC_eHealth	'Luber This graphic illustrates the wide range of multiple health impacts of climate change #CDCGrandRounds http//tco/zWgshayUEi'	Y	96
1/20/2015	CDCgov	Follow @CDC_eHealth today at 1pm ET for live tweeting of #CDCGrandRounds session on birth defects #1in33 http//tco/l8446AZKfN'	Y	36
2/17/2015	DrFriedenCDC	#Polio has no cure #Vaccination the only way to eradicate it #CDCGrandRounds'	N	74
3/12/2015	CDCgov	Incorporating needs of children into emergency preparedness planning is critical Watch #CDCGrandRounds 3/17 1pm ET http//tco/c7EvROV7E4'	Y	69
4/16/2015	CDCgov	#Skincancer is the most common #cancer in US Follow @CDC_Cancer for live tweeting of #CDCGrandRounds 4/21 1PM ET http//tco/BFUFTpBGAP'	Y	34
5/13/2015	CDCgov	Join us on 5/19 at 1PM ET for next #CDCGrandRounds session on the prevention of Aedes mosquitoborne diseases http//tco/ZfBBWHsjyf'	Y	62
6/16/2015	DrFriedenCDC	#Measles deaths could be prevented by administering a simple and safe #vaccine #CDCGrandRounds'	N	47
7/17/2015	CDCgov	Watch #CDCGrandRounds encore webcast 7/21 1PM ET on impact of changing climate w/ update from US @Surgeon_General http//tco/lxZlpc4p33'	Y	26
8/14/2015	CDCgov	Adolescence is a critical stage in which health & safety issues can occur Don't miss next #CDCGrandRounds 8/18 1PM ET http//tco/c7EvRODwfu'	Y	52
9/17/2015	CDCgov	Suicide is preventable & a significant public health issue Watch the latest #CDCGrandRounds video & earn free CE http//tco/shzXUL7hF5'	Y	59
9/29/2015	CDCgov	Epidemics are the world's problem not only the problem of individual countries or regions #CDCGrandRounds http//tco/qihDEHRCvX'	Y	37
10/16/2015	CDCgov	Don't miss the live webcast of #CDCGrandRounds on impact of ecigarettes on the health of our nation 10/20 1PM ET http//tco/c7EvRODwfu'	Y	29
11/10/2015	CDCgov	Join us for the next #CDCGrandRounds session on public health strategies to prevent preterm birth on 11/16 10AM ET https//tco/ksQE0xhBga'	Y	63
12/11/2015	CDCgov	Laboratory work saves lives & protects people Don't miss #CDCGrandRounds on lab safety 12/15 1PM ET https//tco/R68kBQIjdZ'	Y	26

1/15/2016	CDCgov	Modeling can help forecast where infections will spread during outbreaks Don't miss #CDCGrandRounds 1/19 1PM ET https//tco/R68kBQIjdZ'	Y	38
2/12/2016	CDCgov	Chronic fatigue syndrome is a devastating disorder with many symptoms Don't miss #CDCGrandRounds 2/16 1PM ET https//tco/R68kBQIjdZ'	Y	55
3/9/2016	CDCgov	Join us for the next #CDCGrandRounds session on addressing health disparities in early childhood on 3/15 1PM ET https//tco/fux6bf9OK3'	Y	78
4/13/2016	CDCgov	Join us for next session of #CDCGrandRounds on hereditary cancer & genomics 4/19 1PM ET https//tco/R68kBQIjdZ https//tco/UMtbmHSuJx'	N	32
5/17/2016	CDCgov	Stroke is 5th leading cause of death in US & major cause of disability Watch #CDCGrandRounds live today 1PM ET https//tco/R68kBQIjdZ'	Y	53
6/21/2016	CDCgov	Join us for the next #CDCGrandRounds on tracking environmental health data 6/21 1PM ET https//tco/R68kBQIjdZ https//tco/Ona4FstOZ9'	Y	31
7/6/2016	CDCgov	'Join us on 7/12 11 AM ET for a special #CDCGrandRounds to celebrate #CDC70 Anniversary https//tco/R68kBQZUCz https//tco/W5v0Exn78U'	Y	36
7/13/2016	CDCgov	'Join us on 7/19 at 1PM ET for encore session of #CDCGrandRounds on prevention of Aedes mosquitoborne diseases https//tco/QffkaK9VKF'	Y	63
8/16/2016	CDCgov	Today's #CDCGrandRounds discusses strategies to prevent Neonatal Abstinence Syndrome Watch live at 1PM ET https//tco/R68kBQIjdZ'	Y	37
9/16/2016	CDCgov	'Follow @CDC_eHealth on 9/20 1PM ET for live tweeting of #CDCGrandRounds on the importance of newborn screening https//tco/R68kBQZUCz'	Y	26
10/12/2016	CDCgov	'Join us 10/18 1PM ET for our #CDCGrandRounds webcast on new diagnostic tests used to identify infectious diseases https//tco/QztzakWGDz'	Y	51

Table S2. The tweet with the highest number of retweets for each cycle of #VitalSigns and whether it contained a visual cue (an image or a video).

Cycle	Date (m/d/y)	Topic	Twitter Handle	CDC #VitalSigns related Text	Visual cue (Yes/ No)	RT Freq	Top Excluded User	Top Excluded Text	RT Freq
1	3/5/2013	Making Health Care Safer- Stop Infections from Lethal CRE Germs Now	CDCDirector	#VitalSigns sounded alarm about nightmare CRE bacteria Weâ€™ll talk about what we must do to protect patients during #CDCchat 3/25 2PM EDT'	N	5	HamCommFdn	'Terry Cooke talks immigration in #HamOnt with the Drs Gary Warner and @SarahVWayland on the latest @Cable14 #VitalSigns'	3
2	4/2/2013	Preventing Repeat Teen Births	CDCgov	'Teen births have declined for the past two decades but 183 repeat teen births occur/day Learn more http//tco/aDSKzB4ROR #VitalSigns'	N	75	JunaidKausar	'Cover of the 1st #VitalSigns album released in 1989 @SufiSal @RohailHyatt @ShahiHasan @JunaidJamshedPK http//tco/uiq9QamYZY'	20
3	5/7/2013	Hepatitis C	CDC_HIVAIDS	'1 in 4 new HIV infections occur in people 1324 Have a question about HIV? Ask an expert http//tco/xs95z9DXaf #VitalSigns'	N	42	nickhurdmp	'Congrats to @CFTyneWearNland on #vitalsigns initiative Started in Canada now here Helps shape debate on local need and direct local giving'	10
4	6/4/2013	Recipe for Food Safety	CDCgov	'Hot off the press! #Listeria a rare &amp deadly germ is the number 3 cause of #death from germs in foods #VitalSigns http//tco/HKh82keRGJ'	Y	31	sufisal	'Cover of the 1st #VitalSigns album released in 1989 @RohailHyatt @ShahiHasan @JunaidJamshedPK http//tco/uydDrrJWIT'	25

5	7/2/2013	Prescription Painkiller Overdoses	CDCgov	'Prescription painkiller overdoses among women increased fivefold 19992010 #VitalSigns http//tco/XBHUkkNrS1 http//tco/D4ssXYFXPy'	Y	51	imagineCALGARY	'Today is the last day to complete @CalgFoundation #VitalSigns survey http//tco/sha4r4nLsd Rank #yyc on 15 key areas!'	6
6	8/6/2013	Progress on Childhood Obesity	CDCgov	'Good news! After decades of rising childhood obesity rates are now showing small declines in many states #VitalSigns http//tco/7GmtzHVoYP'	Y	74	cfgp_	'FriendsPlease help share a #VitalSigns survey! http//tco/rQKVtnepLj @Ptbo_Canada @kawarthaNOW @wbnptbo @PtboEcDev @ptbochamber #ThankYou!'	8
7	9/3/2013	Preventable Deaths from Heart Disease & Stroke	CDCgov	'Check out #VitalSigns for tips on the ABCS to prevent #HeartDisease &amp #Stroke http//tco/emZEFjxWTK http//tco/23IlLPcr9S'	Y	52	CommFdnsCanada	'Get inspired to do #3things4food by watching our short video on the food situation in Canada http//tco/EjYCutLR8n #vitalsigns'	17
8	11/5/2013	Colorectal Cancer Tests Save Lives	CDCgov	'Colorectal #cancer screening tests saves lives but screening rates remain too low #VitalSigns http//tco/nXaIqZi1bg http//tco/xsTFNTzIgc'	Y	76	YoungChris	'DEC10TH #VitalSigns Will be Available on ITunes Pls download &amp Support! Thx in advance goodMorning http//tco/gvKpbI6hYG'	14
9	1/7/2014	Alcohol Screening and Counceling	CDCgov	'1 in 6 US adults say a health provider has talked w/ them about their alcohol use #VitalSigns http//tco/PVMCx7L2hX http//tco/IfBJnvU1dj'	Y	69	changeequation	'New #VitalSigns data show US elementary students spend only 26 hrs/wk on science #ScienceSOS http//tco/91Dd0FSiNd http//tco/i52fWIRzEd'	22
10	2/4/2014	Child Passenger	CDCgov	'From 20022011 crash deaths among children dropped 43% but 9000+ still	Y	47	sufisal	'Stay tuned! http//tco/J47fHe5XiQ @lasiaffaisal	4

		Safety		died #VitalSigns http//tco/CDCjY2FTEb http//tco/qXWTHJcPBp'				@JunaidJamshedPK don't see anyone getting even close to #vitalsigns &amp #junoon #PakvsInd'	
11	3/4/2014	Making Health Care Safer – Antibiotic Rx in Hospitals: Proceed with	CDCgov	'All hospitals should start an antibiotic stewardship program #SaveAbx #VitalSigns http//tco/XuRA2Z5YS1 http//tco/acsPhSfC07'	Y	198	Mobeen_Ansari	'Selfie level Junaid Jamshed!! #VitalSigns #Dharkan #DilDilPakistan http//tco/Fk5cG2x2Sg'	33
12	4/8/2014	Preventing Pregnancies in Younger Teens	CDCgov	'Health care providers are you providing teen friendly care? Get helpful tips http//tco/m1cJry4Srq #VitalSigns http//tco/rzjeYs0knp'	Y	63	strzel_a	'#VitalSigns http//tco/53LtfkTL3W'	2
13	5/6/2014	Adults with Disabilities	CDCgov	'CDC resource helps connect adults with #disabilities to physical activity programs http//tco/XYI6MgD8lj #VitalSigns http//tco/0s2yDxgPf9'	Y	83	gfriese	'Course for aspiring paramedics? Understanding What Body Is Telling Us @pennopencourses @Coursera! https//tco/GMVjLGSfRN #vitalsigns'	6
14	6/3/2014	Preventing Norovirus Outbreaks	CDCgov	'New #VitalSigns on preventing #norovirus outbreaks Food service industry has key role http//tco/QBkBwv4P6K http//tco/2cPZgs9JF9'	Y	193	RWJF_Live	'MT @RWJF How can we collect #vitalsigns that consider overall wellbeing? Share your thoughts http//tco/MWKG5yv9Z7 http//tco/o8uI0AVTbC'	11
15	7/1/2014	Opioid Painkiller Prescribing	CDCgov	'Overprescribing painkillers is risky to patients and the practice varies by state #VitalSigns http//tco/QzxqbT4yau	Y	78	sufisal	'Lets #endpolio Now Pakistan #VitalSigns #Junoon http//tco/1UYHqO5qev'	39

				http//tco/WvixxhU5y0'					
16	8/12/2014	Children Eating More Fruit Not More Vegetables	CDCgov	'9 in 10 kids don‰Ûªt eat enough vegetables And many of the veggies they do eat are fried white potatoes #VitalSigns http//tco/lTWCqyZKlZ'	Y	91	izahashmi	'"@sufisal Lets #endpolio Now Pakistan #VitalSigns #Junoon http//tco/iY5hPi8ihA"'	16
17	9/9/2014	Reducing Sodium in Children's Diets	CDCgov	'Of schoolage #children teens eat the most #sodium every day See how much http//tco/i9naMkSOY2 #VitalSigns http//tco/A7odOEjyiM'	Y	78	MeWrote	'Junoon reborn! Vital Signs is back We are back into teen ageThanks to @JunaidJamshedPK @sufisal #Junoon #VitalSigns http//tco/SkSwt6giFP'	43
18	10/8/2014	Motor Vehicle Crash Injuries	CDCgov	'Use seat belts on every trip no matter how short Motor vehicle crash injuries are preventable #VitalSigns http//tco/OwbHtYrmVm'	Y	83	VitalSignsPk	'#VitalSigns never broke up The band just got busy working on individual projects It's a common misconception that is clarified'	64
19	11/5/2014	Cervical Cancer is Preventable	CDCgov	'No woman should die of #CervicalCancer Up to 93% of cases could be preventable w/ tests &amp HPV vaccine #VitalSigns http//tco/kE3SRKxkba'	Y	118	AveryDennison	'Saving Panama sea turtles through a unique partnership http//tco/lGpOuE0EWT #followthefrog #vitalsigns @Chiquita http//tco/tojPysPJI9'	12
20	11/25/2014	HIV Care Saves Lives	CDCgov	'Did you know that only 49% of people aged 1824 living with HIV have been diagnosed? http//tco/QPq5bJzHKb #VitalSigns'	Y	80	MelbourneLMCF	'Melbourne 1st Aus capital city participates in international #VitalSigns community wellbeing project http//tco/6B4lPzQuJF #VitalSignsMelb'	15
21	1/6/2015	Alcohol Poisoning	CDCgov	'3 in 4 alcohol poisoning deaths are among middle	Y	228	ISS_Research	'@csa_asc robotics featured in this @CNN @drsanjaygupta	22

		Deaths		aged adults Know the dangers of drinking too much #VitalSigns http//tco/O0vzDM8Kdk'				#VitalSigns segment http//tco/CrAhBznyv7 #OffTheEarthForTheEarth #NeuroArm'	
22	2/3/2015	Secondhand Smoke	CDCgov	26 states and DC have 100% smokefree laws Find out how your state stacks up #VitalSigns http//tco/Ganr3v6Utc http//tco/Tj51mGp8ND'	Y	104	CKXdotorg	'What's the power of data? @CommFdnsCanada is exploring that this year by opening up 10+ years of data from its #VitalSigns program'	9
23	3/3/2015	Trucker Safety	HHSGov	April is #AlcoholAwarenessMonth Know what a "standard drink" in the US is _¾ • http//tco/Zl4rWnHJY2 #VitalSigns http//tco/96LCIXaIRX'	Y	82	TheKingsFund	What needs to change for the care of people with longterm conditions to become truly patientcentred? http//tco/xCsAjXLUr8 #vitalsigns'	49
24	4/7/2015	Preventing Teen Pregnancy	CDC_eHealth	TEENS Did you know that IUDs and implants can prevent pregnancy for 3 to 10 years? #VitalSigns http//tco/7B4LVfO3bc'	Y	35	drsanjaygupta	'in nicoya costa rica people live longer healthier lives what's their secret? find out #vitalsigns 2et on @cnn http//tco/46kxlv8gHM'	195
25	5/5/2015	Hispanic Health	CDCgov	Spanish speaking doctors and community health workers play an important role in Hispanic Health #VitalSigns http//tco/C8QyIqivZQ'	Y	207	drsanjaygupta	'see what @NASA_Astronauts eat aboard the @Space_Station + how they exercise #VitalSigns 230pET today on @CNN @NASA http//tco/VAeK0VmsEY'	38
26	6/2/2015	Preventing Melanoma	CDCgov	Melanoma is the deadliest form of skin cancer killing 9000 people each year #VitalSigns http//tco/rm7FNxlmBA'	Y	97	drsanjaygupta	'Amber has been battling Tourette's for 20+ years see her brain surgery while she's awake at 230p on @CNN #VitalSigns	186

								http//tco/3QmgmoV8id'	
27	7/7/2015	Today’s Heroin Epidemic	CDCgov	From 2002_ÑÐ2013 heroinrelated overdose deaths quadrupled Learn how states can help to save lives http//tco/M6GzfnZ5FJ #VitalSigns'	Y	94	drsanjaygupta	'James Harrison has saved 2+ MILLION babies' lives by donating blood every week for 60 years @CNN #VitalSigns 230pET http//tco/pl7JWxrf6e'	562
28	8/4/2015	Making Health Care Safer – Stop Spread of Antibiotic	CDCgov	Best infection control? Prevention! Wash your hands &amp make sure your providers always wash theirs too #VitalSigns http//tco/HM7WN65oNM'	Y	151	drsanjaygupta	'The race to zero Nigeria's fight to wipe out polio http//tco/dHkhUq9yt3 @cnni #VitalSigns @gatesfoundation @WHO http//tco/ukS1Zkbn1i'	123
29	9/1/2015	Heart Age: Is Your Heart Older Than You	CDCgov	New #VitalSigns report shows 3 in 4 #HeartAttacks &amp #strokes are due to risk factors that increase #HeartAge http//tco/U2fT7QCsB0'	Y	78	RZIMhq	Are there signs of life in todayäó»s culture? Whatäó»s worth paying attention to nowadays? @CamMcAllister7 http//tco/rY6AQRsaa2 #vitalsigns'	17
30	10/6/2015	Hospital Actions Affect Breastfeeding	CDCgov	Babies who are #breastfed have fewer ear respiratory &amp gastrointestinal infections http//tco/UR2mBKgfRP #VitalSigns'	Y	81	spaikin	Toronto now 13th most unaffordable city out of top 86 Avg house price $1m Longest commute times outside New York avg 66 mins #VitalSigns'	30
31	11/3/2015	Safer Foods Saves Lives	CDCgov	Store loyalty card records can help identify foods that made people sick #VitalSigns https//tco/oaYw52RsI6'	Y	51	CamMcAllister7	'How do we explain Christlike love in secular terms? https//tco/DJSCgXA8ey #VitalSigns'	4
32	11/24/2015	Daily Pill Can Prevent	CDCgov	Americans with very high risk for #HIV could benefit	Y	100	cnnhealth	10 diseases you thought were gone https//tco/Uo47Mkbact	14

		HIV		from PrEP a daily pill that prevents HIV https//tco/THlsAXba0C #VitalSigns'				#VitalSigns https//tco/p1K8hXPDuA'	
33	1/5/2016	E-cigarette Ads and Youth	CDCgov	New numbers from #VitalSigns looks at ecigarette marketing and teen ecigarette use https//tco/dh17ByurmW'	Y	71	drsanjaygupta	'Yoga at the Pentagon Why more vets active duty military are turning to an ageold practice @CNN #VitalSigns 230p https//tco/iVds5gmSaU'	90
34	2/2/2016	Alcohol and Pregnancy	CDCgov	Alcohol can cause problems for developing baby at any stage of pregnancy as early as first few weeks #VitalSigns https//tco/ao9rV15cHY'	Y	74	cnnhealth	'Could this glove be the solution to Parkinson's tremors? #VitalSigns https//tco/Ll1s407mam https//tco/01NHeLpwtQ'	37
35	3/3/2016	Making Health Care Safer – Protect Patients from Antibiotic Resistance	CDCDirector	#Antibiotic resistance results when germs are able to resist the effect of drugs designed to kill them #VitalSigns https//tco/ghclul9k7O'	Y	159	cnnhealth	Occasional fasting could help you live longer https//tco/lQq6FR4GA1 #VitalSigns https//tco/YONduF3c7j'	34
36	4/1/2016	Zika and Pregnancy	CDCgov	Pregnant women Avoid travel to areas w/ #Zika If you must travel talk to your Dr first #VitalSigns https//tco/k3NxKsqiSK'	Y	200	TorontoFdn	'From this angle @Drake has some great views of our #VitalSigns Report #TVS2015 ðŸ˜Š https//tco/e4CkD83xho https//tco/iUK1T9ky9Y'	12
37	5/3/2016	ADHD in Young Children	CDCgov	1/3 of children w/ ADHD are diagnosed b/w 25 yrs old Behavior therapy is the rec first step for them #VitalSigns https//tco/sdIvOUYCpT'	Y	68	RZIMhq	New #VitalSigns podcast series on our rapidly changing understanding of identity https//tco/abbfG5bY4n https//tco/JzxjCyBQBX'	21

38	6/7/2016	Legionnaires Disease	CDCgov	#Legionnaire's disease is on the rise Keeping building water systems safe is key to saving lives #VitalSigns https//tco/89PerNQ1rQ'	Y	70	drsanjaygupta	'What can 54 hours in #thecrucible w/ the @USMC teach us about #habits &amp #productivity? #VitalSigns 230pET on @CNN https//tco/wpj1Ike91l'	30
39	7/6/2016	Motor Vehicle Crash Deaths	CDCgov	Find out how the US is doing in motor vehicle safety compared with 19 other highincome countries #VitalSigns https//tco/fjVVAr1baa'	Y	65	drsanjaygupta	'See how 3D printers saved this 4yearold girl's life today on #VitalSigns 230pET on @CNN https//tco/oQBWeWFZt1 https//tco/kP9G3P0IDu'	115
40	8/23/2016	Making Health Care Safer – Think sepsis. Time matters.	CDCgov	Learn sepsis signs and symptoms If suspected immediately seek medical attn Ask äóìCould it be sepsis?äó • #VitalSigns https//tco/zYQzSAYTAl'	Y	87	drsanjaygupta	'FDA trials on a Cuban lung cancer vaccine could start by the end of this year Watch #VitalSigns at 230pET on @CNN https//tco/oqijNLjJYQ'	320
41	9/13/2016	Blood Pressure Control	HHSGov	The latest @CDCgov #VitalSigns addresses #bloodpressure control https//tco/m6Y5zOVdrc https//tco/bpEKFShNb6'	Y	40	CommFdnsCanada	'Kicking off #VitalSigns Week with our National Report Belonging Exploring Connection to Communityâ€¦ https//tco/cZWP7kNbQB'	42
42	10/18/2016	Dental Sealants Prevent Cavities	CDCgov	'Dental sealants prevent decay &amp lower treatment costs They shield teeth from cavities Learn more https//tco/QmqhiY9D7W #VitalSigns'	Y	71	CharityVillage	'Have you read the new #VitalSigns report? Get the highlights here! https//tco/6i5t2fM786 #VSBelongingâ€¦ https//tco/Q1tW7h5G8Q'	9

Freq: Frequency

Table S3. Prevalence ratios of attaching a photo or video on retweet frequency for #CDCGrandRounds over different cycles.

Cycle	Topic	Cycle Date (m/y)	N=Total Tweets in Cycle Corpus n=Manually Coded Tweets	Prevalence Ratio (95% CI)	P-Value
1	Newborn Screening: Improving Outcomes	8/2011	N=22, n=22	1.471 (0.231 - 6.820)	0.642
2	Reducing Severe Traumatic Brain Injuries in the U.S	9/2011	N=22, n=22	0.472 (0.107 - 1.579)	0.260
3	The Science Base for Prevention of Injury Violence	1/2012	N=42, n=30	0.783 (0.297 - 1.926)	0.605
4	Preventing 1 Million Heart Attacks and Strokes by 2017: the Million Hearts Initiative	2/2012	N=49, n=30	0.651 (0.01 - 2.792)	0.596
5	Preventing Excessive Alcohol Use: What Public Health Can Do	3/2012	N=6, n=6	0.651 (0.01 - 2.792)	0.997
6	The Growing Threat of Multidrug-Resistant Gonorrhea	5/2012	N=12, n=12	-	-
7	Breaking the Silence – Public Health's Role in Intimate Partner Violence Prevention	6/2012	N=57, n=30	1.462 (0.711- 3.13)	0.312
**8**	**Global Tobacco Control: A Prevention "Best Buy"**	**7/2012**	**N=144, n=30**	**0.406 (0.219 - 0.783)**	**0.005***
**9**	**High-Impact HIV Prevention**	**8/2012**	**N=203, n=30**	**1.694 (1.175 - 2.448)**	**0.005***
10	Explaining the Unexplained – Discovering New Diseases Using Advanced Detection Tools	9/2012	N=82, n=30	0.835 (0.472 - 1.483)	0.535
11	Public Health Approaches to Reducing U.S. Infant Mortality	10/2012	N=121, n=30	0.967 (0.705 - 1.321)	0.832
12	Unsafe Injection Practices in the U.S. Healthcare System	11/2012	N=100, n=30	0.967 (0.523 - 1.841)	0.917
13	Where in health is disability? Public health practices to include people with disabilities	12/2012	N=156, n=30	1.375 (0.991 - 1.911)	0.057
14	Preventing Venous Thromboembolism	1/2013	N=159, n=30	0.797 (0.545 - 1.168)	0.241
15	Reducing the Burden of HPV-associated Cancer and Disease through Vaccination in the US	2/2013	N=147, n=30	0.896 (0.65 - 1.235)	0.502
16	Reducing Teen Pregnancy in the United States	3/2013	N=196, n=30	1.293 (0.801 - 2.065)	0.287
17	Childhood Immunization as a Tool to Address Health Disparities	4/2013	N=208, n=30	1 (0.704 - 1.421)	1
18	Hypertension: Detect, Connect, Control	5/2013	N=88, n=30	1.812 (0.985 - 3.325)	0.0547

19	The Future of Cancer Screening: Public Health Approaches	7/2013	N=12, n=12	2.954 (0.042 - 43.130)	0.472
20	Technology and Health: Aging Safely and More Independently	9/2013	N=106, n=30	0.997 (0.761 - 1.300)	0.982
21	Combating Resistance: Getting Smart About Antibiotics	11/2013	N=9, n=9	0.997 (0.761 - 1.300)	0.998
22	Advanced Molecular Detection	12/2013	N=158, n=30	1.465 (0.944 - 2.278)	0.089
**23**	**Community Water Fluoridation: A Vital 21st Century Public Health Intervention**	**12/2013**	**N=188, n=30**	**1.523 (1.134 - 2.052)**	**0.005***
24	Science Impact	1/2014	N=221, n=30	1.124 (0.816 - 1.548)	0.474
**25**	**Preventing Youth Violence**	**2/2014**	**N=109, n=30**	**1.840 (1.156 - 2.945)**	**0.011***
**26**	**Multidrug-resistant Tuberculosis: Tools for Tackling a New Face of an Old Foe**	**3/2014**	**N=225, n=30**	**2.179 (1.157 - 4.328)**	**0.020***
**27**	**Evidence-based Intervention for Persons with Autism Spectrum Disorder**	**4/2014**	**N=203, n=30**	**1.432 (1.083 - 1.866)**	**0.010***
**28**	**Pre-Exposure Prophylaxis for Prevention of HIV**	**5/2014**	**N=153, n=30**	**1.264 (1.008 - 1.581)**	**0.041***
**29**	**The 25th Anniversary of the Discovery of the Hepatitis C Virus: Looking Back to Look Forward**	**6/2014**	**N=123, n=30**	**1.563 (1.097 - 2.231)**	**0.014***
**30**	**Time for Public Health Action on Infertility**	**8/2014**	**N=150, n=30**	**1.676 (1.265 - 2.219)**	**<0.001***
**31**	**Preventing A Million Heart Attacks and Strokes: A Turning Point for Impact**	**9/2014**	**N=20, n=20**	**1.448 (1.067 - 1.938)**	**0.015***
32	How Pharmacists Can Improve Our Nation’s Health	10/2014	N=185, n=30	0.840 (0.622 - 1.113)	0.239
**33**	**Unusual Transplant-associated Infections: Just How Unusual?**	**11/2014**	**N=147, n=30**	**2.505 (1.394 - 4.578)**	**0.002***
34	Climate Change and Health – From Science to Practice	12/2014	N=100, n=30	1.266 (0.635 - 2.393)	0.483
**35**	**Understanding the Causes of Major Birth Defects: Steps to Prevention**	**1/2015**	**N=34, n=30**	**34.714 (7.662 - 261.591)**	**<0.001***
**36**	**Global Polio Eradication: Reaching Every Last Child**	**2/2015**	**N=53, n=30**	**2.272 (1.089 - 4.884)**	**0.031***
**37**	**Addressing Preparedness Challenges for Children in Public Health Emergencies**	**3/2015**	**N=124, n=30**	**1.990 (1.474 - 2.691)**	**<0.001***
38	Prevention and Control of Skin Cancer	4/2015	N=85, n=30	1.385 (0.728 2.791)	0.338
**39**	**Dengue and Chikungunya in Our Backyard: Preventing Aedes Mosquito-Borne Diseases**	**5/2015**	**N=117, n=30**	**2.786 (1.728 - 4.619)**	**<0.001***

**40**	**Working to Eliminate Measles Around the Globe**	**6/2015**	**N=136, n=30**	**2.938 (2.003 - 4.339)**	**<0.001***
**41**	**Climate Change and Health – From Science to Practice (An Encore Presentation)**	**7/2015**	**N=78, n=30**	**6.538 (3.437 - 12.659)**	**<0.001***
**42**	**Adolescence: Preparing for Lifelong Health and Wellness**	**8/2015**	**N=117, n=30**	**1.479 (0.897, 2.439)**	**0.124***
**43**	**Preventing Suicide: A Comprehensive Public Health Approach**	**9/2015**	**N=29, n=29**	**36.353 (4.869 - 343.845)**	**<0.001***
**44**	**Shifts in Global Health Security: Lessons from Ebola**	**9/2015**	**N=349, n=30**	**2.007 (1.538 - 2.626)**	**<0.001***
45	E-cigarettes: An Emerging Public Health Challenge	10/2015	N=328, n=30	1.105 (0.709 - 1.701)	0.653
46	Public Health Strategies to Prevent Preterm Birth	11/2015	N=20, n=20	-	-
**47**	**Strengthening a Culture of Laboratory Safety**	**12/2015**	**N=167, n=30**	**2.432 (1.640 - 3.642)**	**<0.001***
**48**	**Staying Ahead of the Curve: Modeling and Public Health Decision-Making**	**1/2016**	**N=127, n=30**	**2.782 (1.778 - 4.409)**	**<0.001***
**49**	**Chronic Fatigue Syndrome: Advancing Research and Clinical Education**	**2/2016**	**N=128, n=30**	**2.127 (1.370 - 3.345)**	**<0.001***
50	Addressing Health Disparities in Early Childhood	3/2016	N=238, n=30	1.407 (0.998 - 2.006)	0.055
**51**	**Cancer and Family History: Using Genomics for Prevention**	**4/2016**	**N=134, n=30**	**2.715 (1.810 - 4.094)**	**<0.001***
52	Mind Your Risks and Act FAST to Prevent and Treat Strokes	5/2016	N=92, n=30	1.216 (0.832 - 1.782)	0.314
53	Tracking Environmental Health Data for Public Health Decision Making	6/2016	N=96, n=30	1.036 (0.623 - 1.696)	0.890
**54**	**Seven Decades of Firsts with Seven CDC Directors**	**7/2016**	**N=74, n=30**	**2.230 (1.433 - 3.490)**	**<0.001***
**55**	**Dengue and Chikungunya in Our Backyard: Preventing Aedes Mosquito-Borne Diseases (an Encore Presentation)**	**7/2016**	**N=158, n=30**	**1.657 (1.119 - 2.442)**	**0.011***
**56**	**Primary Prevention and Public Health Strategies to Prevent Neonatal Abstinence Syndrome**	**8/2016**	**N=105, n=30**	**2.591 (1.638 - 4.142)**	**<0.001***
**57**	**Beyond the Blood Spot: Newborn Screening for Hearing Loss and Critical Congenital Heart Disease**	**9/2016**	**N=95, n=30**	**2.720 (1.787 - 4.172)**	**<0.001***
**58**	**Changes in Clinical Diagnostics and Tracking Infectious Diseases**	**10/2016**	**N=125, n=30**	**3.885 (2.227 - 6.812)**	**<0.001***

Table S4. Prevalence ratios of attaching a photo or video on retweet frequency for #Vitalsigns over different cycles.

**Cycle**	**Topic**	**Start Date m/d/y**	**Total Number of Tweets, N**	**Total Number of Tweets Manually Coded to Obtain a Sample of 30 Relevant Tweets, n**	**Manually Coded Relevant Tweets, n**	**Prevalence Ratio (95% Confidence Interval)**	**P-value**
1	Making Health Care Safer- Stop Infections from Lethal CRE Germs Now	3/5/2013	96	*		-	-
2	Preventing Repeat Teen Births	4/2/2013	304	37	30	0.768 (0.192, 2.383)	0.671
3	Hepatitis C	5/7/2013	221	32	30	1.07 (0.738, 1.544)	0.717
4	Recipe for Food Safety	6/4/2013	234	36	30	2.725 (1.411, 5.193)	**0.002***
5	Prescription Painkiller Overdoses	7/2/2013	218	43	30	33.514 (8.715, 133.357)	**<0.001***
6	Progress on Childhood Obesity	8/6/2013	359	39	30	6.107 1.515, 19.471)	**0.004***
7	Preventable Deaths from Heart Disease & Stroke	9/3/2013	1628	44	30	2.893 (1.122, 6.927)	**0.020***
8	Colorectal Cancer Tests Save Lives	11/5/2013	1284	43	30	5.986 (0.596, 30.225)	0.0558
9	Alcohol Screening and Counceling	1/7/2014	315	36	30	3.093 (1.097, 7.471)	**0.019***
10	Child Passenger Safety	2/4/2014	235	36	30	3.089 (1.344, 6.853)	**0.006***

11	Making Health Care Safer – Antibiotic Rx in Hospitals: Proceed with	3/4/2014	393	32	30	1.03 (0.518, 1.915)	0.928
12	Preventing Pregnancies in Younger Teens	4/8/2014	213	34	30	4.567 (1.348, 12.965)	**0.007***
13	Adults with Disabilities	5/6/2014	256	34	30	1.786 (0.727, 3.892)	0.169
14	Preventing Norovirus Outbreaks	6/3/2014	197	36	30	29.536 (1.330, 326.283)	**0.007***
15	Opioid Painkiller Prescribing	7/1/2014	304	35	30	1.929 (0.919, 3.819)	0.068
16	Children Eating More Fruit Not More Vegetables	8/12/2014	219	33	30	1.735 (0.833, 3.354)	0.118
17	Reducing Sodium in Children's Diets	9/9/2014	1227	38	30	2.024 (0.736, 4.684)	0.129
18	Motor Vehicle Crash Injuries	10/8/2014	723	55	30	3.432 (0.496, 17.912)	0.156
19	Cervical Cancer is Preventable	11/5/2014	210	39	30	1.439 (0.175, 5.655)	0.662
20	HIV Care Saves Lives	11/25/2014	269	33	30	1.785 (0.731, 3.829)	0.164
21	Alcohol Poisoning Deaths	1/6/2015	199	42	30	5.279 (1.450, 15.437)	**0.005***
22	Secondhand Smoke	2/3/2015	256	30	30	3.562 (2.011, 6.073)	**<0.001***
23	Trucker Safety	3/3/2015	252	95	30	10.270 (2.992, 37.010)	**<0.001***
24	Preventing Teen Pregnancy	4/7/2015	207	31	30	1.923	**0.035***

						(1.028, 3.483)	
25	Hispanic Health	5/5/2015	193	42	30	2.852 (1.068, 6.729)	**0.023***
26	Preventing Melanoma	6/2/2015	173	58	30	2.298 (0.604, 6.522)	0.158
27	Today's Heroin Epidemic	7/7/2015	238	36	30	2.279 (1.140, 4.258)	**0.014***
28	Making Health Care Safer – Stop Spread of Antibiotic	8/4/2015	284	40	30	3.678 (1.484, 8.037)	**0.002***
29	Heart Age: Is Your Heart Older Than You	9/1/2015	287	33	30	1.908 (0.720, 4.366)	0.153
30	Hospital Actions Affect Breastfeeding	10/6/2015	295	42	30	1.256 (0.534, 2.614)	0.568
31	Safer Foods Saves Lives	11/3/2015	151	48	30	3.014 (1.042, 7.509)	**0.026***
32	Daily Pill Can Prevent HIV	11/24/2015	212	32	30	1.714 (0.165, 7.590)	0.552
33	E-cigarette Ads and Youth	1/5/2016	297	42	30	1.694 (0.834, 3.206)	0.122
34	Alcohol and Pregnancy	2/2/2016	295	62	30	2.651 (0.931, 6.454)	**0.045***
35	Making Health Care Safer – Protect Patients from Antibiotic Resistance	3/3/2016	239	46	30	2.293 (0.867, 5.149)	0.063
36	Zika and Pregnancy	4/1/2016	219	30	30	1.047 (0.566, 2.078)	0.889
37	ADHD in Young Children	5/3/2016	449	43	30	1.957 (0.528, 5.667)	0.254
38	Legionnaires Disease	6/7/2016	282	71	30	2.787	0.063

						(0.827, 7.568)	
39	Motor Vehicle Crash Deaths	7/6/2016	325	85	30	6.686 (1.345, 28.132)	**0.011***
40	Making Health Care Safer – Think sepsis. Time matters.	8/23/2016	236	33	30	3.516 (1.680, 6.859)	**<0.001***
41	Blood Pressure Control	9/13/2016	827	30	30	1.27 (0.761, 2.063)	0.346
42	Dental Sealants Prevent Cavities	10/18/2016	194	**		-	-

Footnotes:

*Only 7 of the tweets were relevant to the CDC Vital Signs report. Therefore, we decided to exclude that cycle for the analysis here.

**The prevalence ratio could not be computed due to all the tweets in Cycle 42 containing a photo.
